# Daily Stressor-Related Negative Mood and its Associations with Flourishing and Daily Curiosity

**DOI:** 10.1007/s10902-021-00404-2

**Published:** 2021-05-08

**Authors:** Alexandra Drake, Bruce P. Doré, Emily B. Falk, Perry Zurn, Danielle S. Bassett, David M. Lydon-Staley

**Affiliations:** 1 Department of Bioengineering, School of Engineering and Applied Science, University of Pennsylvania, 3620 Walnut Street, Philadelphia, PA 19104-6321, USA; 2 Annenberg School for Communication, University of Pennsylvania, 3620 Walnut Street, Philadelphia, PA 19104-6321, USA; 3 Department of Psychology, University of Pennsylvania, Philadelphia, PA 19104, USA; 4 Wharton Marketing Department, University of Pennsylvania, Philadelphia, PA 19104, USA; 5 Department of Philosophy, American University, Washington, DC 20016, USA; 6 Department of Electrical and Systems Engineering, School of Engineering and Applied Science, University of Pennsylvania, Philadelphia, PA 19104, USA; 7 Department of Neurology, Perelman School of Medicine, University of Pennsylvania, Philadelphia, PA 19104, USA; 8 Department of Psychiatry, Perelman School of Medicine, University of Pennsylvania, Philadelphia, PA 19104, USA; 9 Leonard Davis Institute of Health Economics, University of Pennsylvania, 3620 Walnut Street, Philadelphia, PA 19104-6321, USA

**Keywords:** Flourishing, Stressor exposure, Curiosity, Negative mood, Daily diary

## Abstract

There are pronounced individual differences in the extent to which affective responses are associated with daily stressor exposure. These individual differences have implications for health and well-being. We use 21 days of daily diary data in 167 participants (mean age = 25.37, *SD* = 7.34; 81.44% women) and test (1) the moderating effect of flourishing on daily stressor-related negative mood and (2) the moderating effect of daily curiosity on daily stressor-related negative mood. Results indicate that people high in flourishing show lower stressor-related negative mood and that stressor-related negative mood is higher than usual on days of lower than usual curiosity. Together, these findings extend a large body of work indicating associations between stressor-related negative mood and both psychopathology and poor physical health to trait and state markers of well-being.

## 1 Introduction

Stressors come in a variety of forms, including major life events such as bereavement or marital separation (Holmes & Rahe, 1967) and everyday hassles such as arguments with family members or deadlines at work (Almeida, 2005). Although seemingly minor when contrasted with major events, daily stressors are frequent (Almeida et al., 2002; Stawski et al., 2008), have immediate and direct effects on affective functioning (Fosco & Lydon-Staley, 2017; Stawski et al., 2013), and their effects on mental and physical health can accumulate over time (Chiang et al., 2018; Parrish et al., 2011). People are generally reactive when exposed to daily stressors, showing higher negative mood on days when a stressor occurs compared to days when they are not exposed to stressors (Bolger et al., 1989). However, there are pronounced individual differences in the extent to which negative affective responses are associated with daily stressor exposure (Gunthert et al., 1999; Koffer et al., 2019). These individual differences in stressor-related negative affect (Stawski et al., 2019), a term that captures the association between fluctuations in stressor exposure and fluctuations in negative affect, are associated with both concurrent and long-term mental and physical health difficulties (Charles et al., 2013; Leger et al., 2018; Myin-Germeys et al., 2003; Peeters et al., 2003). Here, we examine daily stressor-related negative mood and examine its associations with flourishing and daily curiosity.

### 1.1 Daily Stressors and Flourishing

Stress is a process that beings with exposure to a stimulus which, when perceived as harmful or threatening, results in a stress response (Smyth et al., 2018). Such stimuli include daily stressors, often taking the form of hassles that people experience in daily (Koffer et al., 2017). The study of daily stressors benefits from research designs that incorporate intensive repeated measures *in situ* to capture day-to-day fluctuations in stressor exposure (Bolger et al., 2003; Smyth & Stone, 2003). With these time series data, researchers can quantify how days of exposure to stressors are associated with an individual’s level of negative affect relative to days of no stressor exposure (Almeida, 2005). Studies that repeatedly assess individuals’ exposure to common stimuli encountered in daily life, such as having an argument with someone and having something bad happen to a close friend or relative (Almeida et al., 2002; Koffer et al., 2019; Leger et al., 2018), find that exposure to such stimuli elicits robust, same-day emotional and physiological responses (Bolger et al., 1989; Sin et al., 2015).

Exposure to daily stressors, then, tends to elicit a negative affect response. The most robust negative affect response to a stressor is likely when there is an imbalance between perceived demands elicited by the stressor and perceived capacity to adapt to those demands (Lazarus & Folkman, 1984). Previous work has identified a range of between-person factors that are associated with greater stressor-related negative affect in the form of an exacerbated affective response to stressor exposure. There is substantial work associating stressor-related negative affect with between-person differences in poor mental and physical health (Charles et al., 2013; Leger et al., 2018; Myin-Germeys et al., 2003; Peeters et al., 2003). Much less is known about the extent to which stressor-related negative affect is associated with well-being. Due to the many resources that accompany well-being, it is likely that well-being has implications for one’s perceived capacity to adapt to life’s daily hassles.

Flourishing refers to an individual’s perception that their life is going smoothly. It is a combination of feeling good (hedonic or subjective well-being) and functioning well (eudaimonic or psychosocial well-being; Datu, 2018; Huppert & So, 2013; Keyes, 2002; Ryff & Singer, 2009). Flourishing encompasses many components, including purpose in life, engagement, competence, positive relationships, and contribution to the well-being of others, thus representing a more comprehensive concept than well-being measures such as life satisfaction (de la Fuente et al., 2019; Diener et al., 2010). The many emotional, psychological, and social resources associated with flourishing likely impact how individuals high in flourishing respond to life’s daily hassles given that robust negative affect responses to stressors are less likely in the context of substantial resources that can be used to adapt to stressor-related demands (Lazarus & Folkman, 1984). For example, people high in flourishing experience high levels of social support (Abdollahi et al., 2018; Schotanus-Dijkstra et al., 2016) and may have people they can turn to in order to help manage daily hassles. Further, people high in flourishing score high on coping competence (Akin & Akin, 2015; Rahim, 2019), exhibit less venting, behavioral disengagement and self-blame, and more planning, positive reframing, and active coping than people who are low in flourishing (Faulk et al., 2013; see also Freier et al., 2018). As such, we hypothesize that individuals high in flourishing will exhibit lower stressor-related negative mood relative to individuals low in flourishing.

### 1.2 Daily Curiosity as a Moderator of Daily Stressor-Related Negative Mood

Associations between daily stressor-related affect and between-person characteristics such as psychopathology and well-being indicate who tends to show strong affective associations with experiences of stressor exposure. It is also important to consider when a given person may experience a stronger than usual association between affect and stressor exposure. One time-varying resource that people may leverage to cope with daily stressors is a capacity to approach stressful experiences with an attitude of curiosity. Curiosity is defined in many ways throughout the literature (e.g., Grossnickle, 2014; Zurn & Shankar, 2020). Curiosity as we examine it here, entails actively seeking opportunities for new experiences and exhibiting a willingness to embrace the novel, uncertain, and unpredictable nature of everyday life (Kashdan et al., 2009). This helps people to effectively cope with novelty and uncertainty. Indeed, during states of curiosity, people show a remarkable ability to tolerate potential sources of distress, being less defensive, less reactive to discomfort and difficulties, and more tolerant of uncertainty (Denneson et al., 2017; Kashdan et al., 2013; Silvia, 2005). This is reflected in the measurement of curiosity. Items emphasize tendencies to view challenging situations as opportunities to grow and learn (Kashdan et al., 2009). Consequently, we asked whether states of curiosity might moderate the association between daily stressor exposure and negative mood.

### 1.3 The Present Study

The goals of this study were twofold. We aimed to test the extent to which a between-person factor, flourishing, and a within-person factor, daily fluctuations in curiosity, were associated with stressor-related negative mood. These goals can by summarized with two main hypotheses:

#### Hypothesis 1

We hypothesized a negative association between daily stressor-related negative mood and flourishing, such that daily stressor-related negative mood would be lower in people who are high versus low in flourishing.

#### Hypothesis 2

We hypothesized that (a) the association between exposure to a daily stressor and negative mood would be positive, and that (b) daily curiosity would moderate daily stressor-related negative mood such that the association between exposure to a daily stressor and negative mood would be stronger on days of lower versus higher than usual curiosity.

## 2 Method

We use data from the Knowledge Networks Over Time (KNOT) study, an intensive longitudinal study designed to provide insight into day-to-day intraindividual variability across a range of domains of functioning, in particular curiosity (Lydon-Staley et al., 2019b; Lydon-Staley et al., 2019a). Data and code used in the manuscript are available at this OSF page: https://osf.io/kjz7g/.

### 2.1 Participants

Participants were 167 individuals (136 women, 29 men, 2 other gender) recruited through poster, Facebook, Craigslist, and university research site advertisements in Philadelphia and the surrounding university community. Individuals were eligible if they met 4 criteria: (1) aged between 18 and 65 years; (2) consistent access to a computer connected to the internet at home; (3) willingness to complete 21 consecutive days of surveys; (4) willing to visit the research laboratory for 1 h. Participants were aged between 18.21 and 65.24 years (*M* = 25.37, *SD* = 7.34), and identified as white (49.10%), African American/Black (8.38%), Asian (23.35%), Hispanic/Latino (4.79%), multiracial (6.59%), other (5.39%), and missing information (2.40%). Participants identified as bisexual (7.78%), gay (4.19%), heterosexual (79.04%), lesbian (1.20%), other (5.99%), and missing information (1.80%). Participants reported a yearly family income ranging from ‘under $20,000’ to ‘$200,000 or more’ (*Modal income* = ‘$20,000–$49,000’). Participants’ education spanned less than a high school degree (0.60%), high school degree (8.98%), associate’s degree or some college but no degree (30.54%), college degree (37.72%), graduate or professional training (20.96%), or missing information (1.20%).

### 2.2 Procedure

Interested participants encountered study advertisements and were directed to a website with study information and a consent form. After confirming that participants met inclusion criteria, participants were contacted by telephone with a description of the study and an opportunity to assent or decline participation. If individuals assented, an email was sent with a baseline survey containing demographic questionnaires and the flourishing scale used in the present study. The baseline survey also contained additional scales that were not the focus of the present study, such as alcohol use and physical activity, but can be found in other work using these data (Lydon-Staley et al., 2019b; Lydon-Staley et al., 2019a). Once the baseline survey was completed, participants visited the laboratory. At the laboratory, participants completed additional questionnaires, received training in the daily assessment protocol, and were guided through the installation of an application necessary for an internet browsing study component that we do not report on in the present manuscript (but see Lydon-Staley et al., 2019c for details).

Following the laboratory visit, a 21 day diary assessment protocol was initiated. The 21 day diary assessment consisted of two components. The first was a daily diary consisting of survey questionnaires that took approximately 5 min to complete. The second came immediately after the daily diary component and was a 15 min internet browsing task that we do not report on in the present manuscript. Links to the daily assessments were emailed to participants at 6:30 PM each evening. Participants who requested reminders received a text message at 6:40 PM to notify them that survey links had been emailed. Participants were instructed to complete the daily assessments before going to bed, but links remained open until 10:00 AM the next morning. In cases where participants completed the surveys the following morning, they were instructed to report as if they were completing the survey on the previous evening. Daily questionnaires took approximately 5 min to complete.

Participants were compensated with gift cards to Amazon.com at each study phase. Participants received $25 after completing the baseline assessment and the laboratory visit. For the daily assessment, completion was incentivized by making participant payment contingent on completion: completion of 3, 4, 5, 6, and 7 surveys each week was compensated with gift cards worth $10, $15, $20, $25, and $35, respectively. Participation was further incentivized through a raffle for which an iPad mini was available as a prize. Completion of 7 surveys each week resulted in one entry into the raffle drawing. Compensation was provided as incentives increase survey completion rates (Bonke & Fallesen, 2010) and are recommended by daily diary researchers to increase the feasibility of daily diary designs (Janssens et al., 2018). We note that the use of incentives can impact who decides to participate in a study and how people respond in a study (Hsieh & Kocielnik, 2016) and that compensation was based on survey completion but not any particular responses to the surveys.

### 2.3 Measures

The present study used participants’ reports of demographic and trait characteristics from the baseline surveys and their daily diary reports. Means, standard deviations, and correlations among the measures used below can be found in Table 1.

#### 2.3.1 Trait Flourishing

Flourishing was measured using an 8-item flourishing scale (Diener et al., 2010). The flourishing scale contains items related to important aspects of functioning, including positive relationships, feelings of competence, and having meaning and purpose in life. Flourishing scale items are answered on a 1 (“Strong Disagreement”) to 7 (“Strong Agreement”) scale. The mean value of all 8 items was taken as a measure of flourishing, with higher values indicating relatively higher levels of flourishing. The scale demonstrated high internal consistency in the current sample (α= 0.90).

#### 2.3.2 Daily Negative Mood

The average of two items, each adapted from the Profile of Mood States (Terry et al., 2003), was used to measure depressed mood (“How much of the time today did you feel _?” “depressed” and “sad or blue”), anxious mood (“anxious” and “worried”), and angry mood (“angry” and “annoyed”). Participants rated their mood using a slider ranging from 0 (‘None of the time”) to 10 (“All of the time”) in 0.1 increments (i.e., 0.0, 0.1, 0.2, …, 10.0). Reliability analyses indicated that the scales were satisfactory in reliably capturing both systematic within-person change in depressed mood (*R*_*c*_ = 0.82), anxious mood (*R*_*c*_ = 0.79), and angry mood (*R*_*c*_ = 0.77), and between-person differences in depressed mood (*R*_1*F*_ = 0.84), anxious mood (*R*_1*F*_ = 0.78), and angry mood (*R*_1*F*_ = 0.69). These daily scales have been used in previous experience-sampling studies (Lydon-Staley et al., 2019d; Lydon-Staley et al., 2019a). Scores from the three scales were averaged to create a daily negative mood variable.

#### 2.3.3 Daily Stressors

Daily stressor exposure was measured through the daily diary component of the study. The measure was based on previous experience-sampling work on stress in daily life (Koffer et al., 2019). Participants were asked to “Rate whether you did or did not experience these sources of stress today” and noted that they either “Did not experience” or “Did experience” the following events: “interpersonal tensions”, “home”, “work/education”, “finances”, “health/accident”, “events that happened to others”, “being evaluated”, “other”. These items were adapted from the Daily Inventory of Stressful Events (Almeida et al., 2002). Reports of daily stress events were common, with the average participant experiencing a stressor on 84% of their daily diary days (mean proportion of days with a stressor = 0.84, SD = 0.20). In line with previous work on daily stressor-related affect (Koffer et al., 2016, 2019) we created two variables from this measure. We summarized across the sources of stressful events to create a time-varying, binary daily stressor variable indicating whether (1) or not (0) any stressor occurred on that day. We also created a person-level stressor exposure variable by computing the proportion of days that a stressor occurred across the 21 days of the daily diary protocol.

#### 2.3.4 Daily Curiosity

Daily curiosity was measured during the daily diary component of the study using 2-items from the Curiosity and Exploration Inventory II (CEI-II; Kashdan et al., 2009) that have been used in previous studies of daily curiosity (e.g., Kashdan et al., 2013). Participants responded to two items. One item, “Today, I viewed challenging situations as an opportunity to grow and learn”, was derived from the embracing subscale of the CEI-II and was designed to provide insight into a willingness to embrace the novel, uncertain, and unpredictable nature of everyday life. A second item, “Everywhere I went today, I was out looking for new things or experiences”, was derived from the stretching subscale of the CEI-II and was designed to provide insight into the motivation to seek out knowledge and new experiences. We used a smaller number of items (*n* = 2) relative to previous daily diary work using this scale (*n* = 4; Kashdan et al., 2013) in order to reduce participant burden during the daily diary component of the study. Participants responded to the items on a slider ranging from 0 (“Not at all”) to 10 (“Very”) in increments of 0.1. A repeated measures correlation (Bakdash et al., 2017) indicated that the two items were moderately correlated, r_rm_ = 0.45, *p* < 0.001. Responses across the items were averaged to form a daily curiosity scale, with higher values indicating higher levels of curiosity. Reliability analysis indicated that the scale was satisfactory in reliably capturing both systematic within-person change in stress (*R*_*c*_ = 0.62) and between-person differences in stress across the daily diary period (*R*_1*F*_ = 0.81).

### 2.4 Data Analysis

Two main questions guided our analyses which we parsimoniously analyzed in one statistical model. We examined the extent to which flourishing moderated the association between today’s stressor exposure and negative mood. Second, we examined the moderating effect of today’s curiosity on the association between today’s stressor exposure and negative mood.

#### 2.4.1 Multilevel model

A multilevel model framework was adopted to accommodate the nested nature of the intensive repeated measures data (21 days nested within 167 persons). To facilitate a focus on within-person associations between today and yesterday’s stress and negative mood, the predictor variables were parameterized to separate within-person and between-person associations (Curran & Bauer, 2011). In addition to allowing us to focus on within-person associations, this approach allowed us to account for between-person differences in time varying constructs (e.g., daily negative mood, daily curiosity, daily stressor exposure) across the 21 days of the daily diary by incorporating information about the usual levels of these constructs at the second level of the multilevel model. This parameterization was achieved by creating time-invariant (between-person) and time-varying (within-person) versions of the predictor variables (Bolger & Laurenceau, 2013). We calculated the time-invariant, between-person variable for usual curiosity as the grand-mean centered individual mean score of daily curiosity reports across all days in the daily diary study. Participants with a positive value on this variable had greater than usual levels of curiosity throughout the study compared with other participants in the sample. We calculated a time-varying, within-person version of the daily curiosity variable as deviations from these between-person, usual curiosity, means. Thus, zero on this within-person variable indicated days of usual levels of curiosity, negative values indicated days of less than usual curiosity, and positive values indicated days of more than usual curiosity for each individual. For the stressor exposure variable, we follow Koffer et al. (2019) and compute usual stressor exposure as the proportion of study days on which a stressor occurred (grand-mean centered so that 0 for the usual stressor exposure value for the prototypical participant in the sample) and use a binary daily stressor variable indicating whether (1) or not (0) any stressor occurred on that day.

The variables were then included in a multilevel model to estimate associations between daily reports of stressor exposure and negative mood and the extent to which these associations were moderated by person-to-person differences in flourishing and day-to-day differences in curiosity. At Level 1 (day-level variables) the formal model was constructed as:

(1) $$NegativeMood_{it}=\beta_{0i}+\beta_{1i}Today^{\prime }sStressorExposure_{it}+\beta_{2i}Today^{\prime }sCuriosity_{it}+\beta_{3i}\text{Toda}y^{\prime }sStressorExposure*Today^{\prime }sCuriosity_{it}+e_{it}$$

where ${\text{β}}_{0}$ is the intercept, indicating the average level of negative mood for the prototypical participant (all predictors were sample-mean centered); ${\text{β}}_{1\text{i}}$ indicates within-person differences in today’s negative mood associated with within-person differences in today’s stressor exposure (i.e., stressor-related negative mood); ${\text{β}}_{2\text{i}}$ indicates within-person differences in today’s negative mood associated with within-person differences in today’s curiosity; ${\text{β}}_{3\text{i}}$ tests for an interaction between today’s stressor exposure and today’s curiosity. Lastly, ${\text{e}}_{\text{it}}$ are autocorrelated day-specific residuals (AR1).

Person-specific intercepts and associations (from Level 1) were specified (at Level 2) as:

(2) $$\begin{array}{l} \beta_{0}=\gamma_{00}+\gamma_{01}Flourishing_{i}+\gamma_{02}UsualStressorExposure_{i}+\gamma_{03}UsualCuriosity_{i}+u_{0i},\\ \beta_{1}=\gamma_{10}+\gamma_{11}Flourishing_{i}+u_{1i}\\ \beta_{2}=\gamma_{20}+u_{2i},\\ \beta_{3}=\gamma_{30}, \end{array}$$

where γ denotes a sample-level parameter and u denotes residual between-person differences that may be correlated, but are uncorrelated with ${\text{e}}_{\text{it}}$. Parameters $\gamma_{01}$ and $\gamma_{02}$ indicate how between-person differences in the average level of negative mood across the daily diary protocol were associated with flourishing and usual levels of stressor exposure. Parameter $\gamma_{11}$ tests the moderating effect of trait flourishing on the association between today’s stressor exposure and today’s negative mood. The multilevel model was fit with nlme in R (Pinheiro et al., 2012) and incomplete data were treated using the assumption of being missing at random. We further probed significant interactions using the Johnson-Neyman technique (Bauer & Curran, 2005). Results were robust to the inclusion of age, gender, day of study, and previous day’s stressor exposure as covariates. In additional analyses, no significant interactions emerged between yesterday’s stressor exposure and flourishing or day’s curiosity. A repeated measures correlation (Bakdash & Marusich, 2017) between today and yesterday’s stressor exposure indicated a significant and small correlation, *ρ*_*rm*_(2567) = 0.13, *p* < 0.001.

**Fig. 1:**
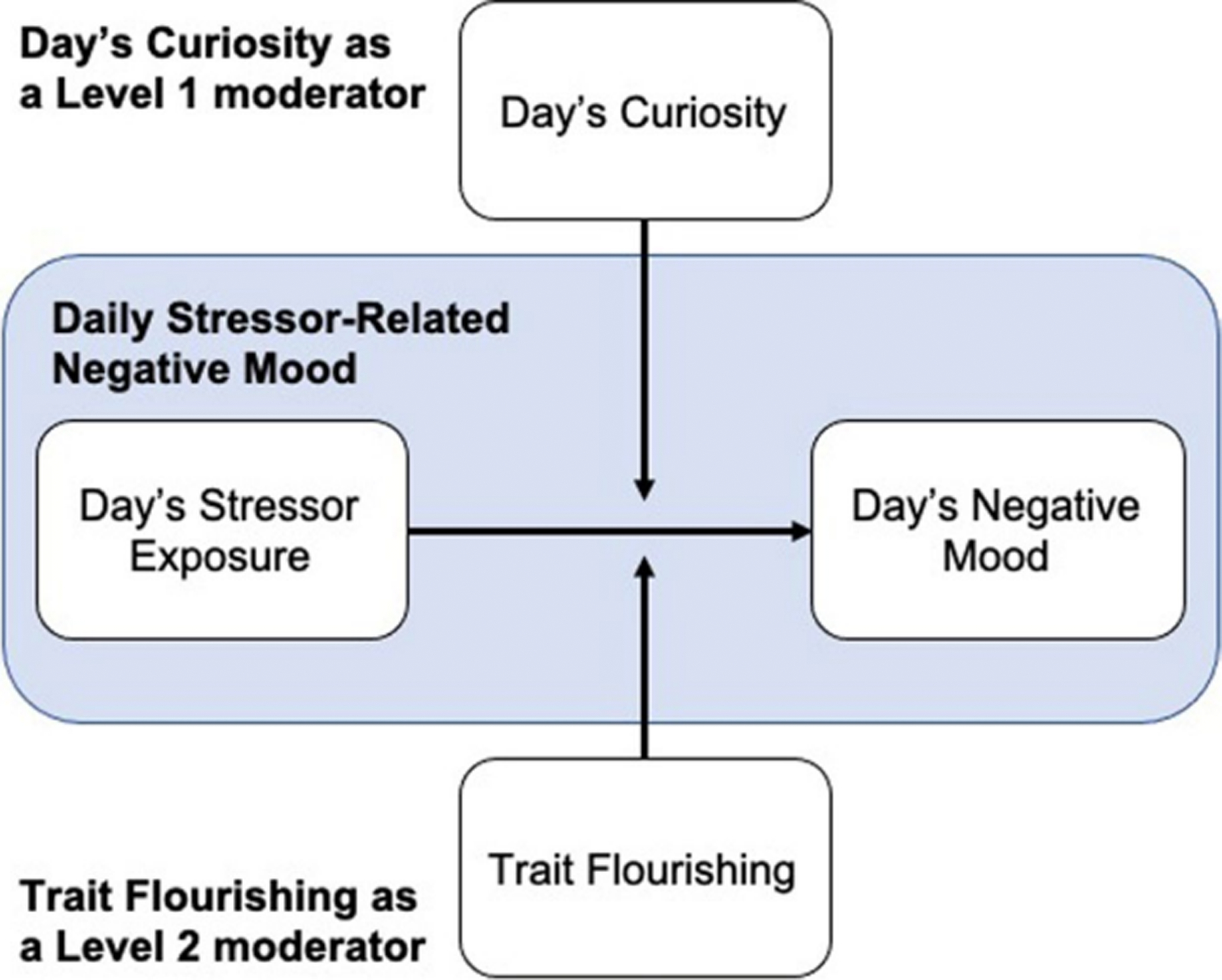
Conceptual model. Daily stressor-related negative mood captures the association between day’s stressor exposure and day’s negative mood and is depicted as an arrow pointing from day’s stressor exposure to day’s negative mood. We hypothesize that the association between day’s stressor exposure and day’s negative mood will be positive and that this association will be moderated by day’s curiosity, such that the association between day’s stressor exposure and day’s negative mood will be stronger on days of lower versus higher than usual curiosity. We depict this moderating role of day’s curiosity with a directed arrow towards the association between day’s stressor exposure and day’s negative mood. We additionally hypothesize a negative association between daily stressor-related negative mood and flourishing, such that daily stressor related negative mood will be lower in people who are high versus low in flourishing. We depict this moderating role of flourishing with a directed arrow towards the association between day’s stressor exposure and day’s negative mood. We highlight that day’s curiosity is a level 1 (day-level) moderator and that trait flourishing is a level 2 (trait-level) moderator

We conducted a power analysis using a different daily diary dataset (Lydon-Staley et al., 2019d) from a sample of 151 participants and 21 days of data. The sample was powered to detect a within-person association between two affect variables (happiness and depression; stress was not measured in that previous data) in 95% of 1000 simulated samples (Bolger & Laurenceau, 2013). This suggests that our larger study (*n* = 167 also with 21 days of data) was sufficiently powered to detect stressor-related negative mood associations. Further, previous daily diary studies of stress-mood associations have included as few as 6 (Stawski et al., 2008) and as many as 30 (Schilling & Diehl, 2014) end of day assessments per person. Our use of 21 days, while more burdensome to participants than completing a protocol using fewer days, ensured relative high power to detect stress-related negative mood associations given that detection is influenced by the number of assessments (Fig. 1).

## 3 Results

We provide descriptive statistics for the variables used in the analyses in Table 1. Out of a possible total of 3507 daily diary days (21 days × 167 participants), 3141 (89.56%) were available. The number of daily diary days available per participant ranged from 11 to 21 (*M* = 18.81, *SD* = 2.75). No significant correlations emerged between key study variables (those listed in Table 1) and the number of days of data obtained from each participant (all *p*-values > 0.05).

### Hypothesis 1: Associations Between Flourishing and Daily Stressor-Related Negative Mood

**Fig. 2:**
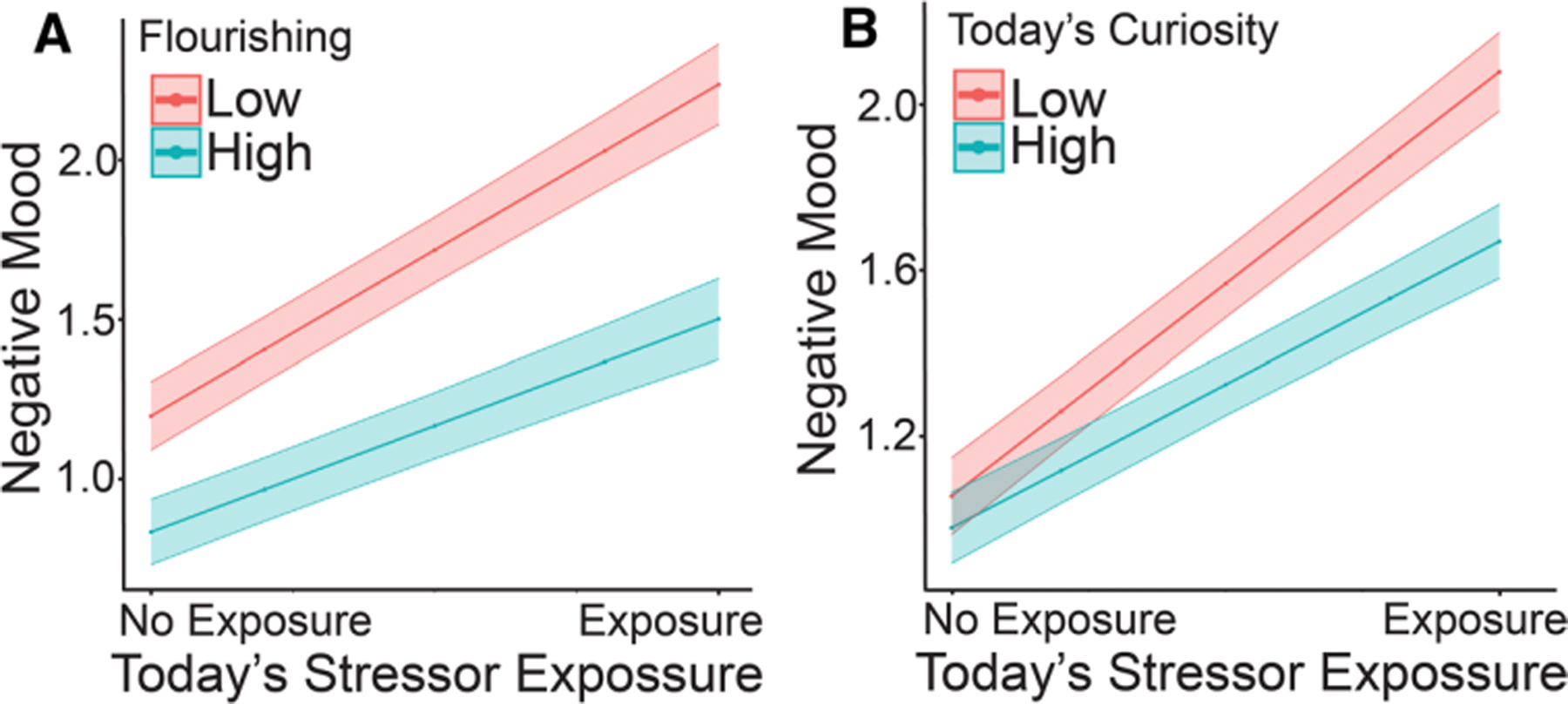
Results of analyses examining associations between flourishing, daily curiosity, and stressor-related negative mood. **a** On days when participants report stressor exposure (x-axis), negative mood (y-axis) is higher than on days of no stressor exposure. The association between today’s stressor exposure and negative mood is attenuated at high values of flourishing. **b** On days when participants report stressor exposure (x-axis), negative mood (y-axis) is higher than on days of no stressor exposure. The association between today’s stressor exposure and negative mood is attenuated on days when participants report being more curiosity than usual. Low and high values of flourishing and day’s curiosity represent + 1/ – 1 standard deviation around the sample average

We tested the hypothesis that daily stressor-related negative mood would be lower in people high in flourishing. Results from the multilevel model are shown in Table 2. A significant interaction emerged between today’s stressor exposure and flourishing ($\gamma_{11}=-0.23$, *p* = 0.01). Considering the interaction further, we found that the association between today’s stressor exposure and negative mood was significant and positive below values of 2.08 on the flourishing scale (centered such that values below 0 indicate scores lower than the average person in the sample). As shown in Fig. 2a, the slope between today’s stressor exposure and negative mood was steeper at lower values of flourishing.

### Hypothesis 2: Today’s Curiosity as a Moderator of Daily Stressor-Related Negative Mood

We hypothesized that daily curiosity would moderate daily stressor-related negative mood such that the association between daily stressor exposure and negative mood would be higher on days of lower than usual curiosity relative to days of higher than usual curiosity. Results of the multilevel model examining the moderating effect of today’s curiosity on the association between daily stressor exposure and negative mood are shown in Table 2. There was a significant interaction between today’s stressor exposure and today’s curiosity ($\gamma_{51}=-0.10$, *p* < 0.001). Considering the interaction further showed that the association between today’s stressor exposure and negative mood was significant for values of today’s curiosity below 5.12 (day’s curiosity was centered such that values below 0 indicated days of lower than usual curiosity). As shown in Fig. 2b, on days of higher than usual curiosity, the association between today’s stressor exposure and negative mood was attenuated.

## 4 Discussion

Daily stressors that we encounter in our daily lives are frequent, have immediate and direct effects on affective function, and can affect physical and mental health. Here, we used a daily diary design to test two hypotheses: (1) participants high in flourishing will exhibit smaller daily stressor-related negative mood relative to participants low in flourishing; (2) daily stressor-related negative mood will be higher on days of lower than usual curiosity relative to days of higher than usual curiosity. We find support for each of these hypotheses which we further detail below.

We observed that the association between today’s stressor exposure and negative mood was smaller in participants high in flourishing relative to those low in flourishing. Flourishing may moderate daily stressor-related negative mood associations through a variety of means. Robust negative affective responses to stressors are especially likely when individuals perceive an imbalance between the perceived demands associated with a stressor and their perceived capacity to adapt to those demands (Lazarus & Folkman, 1984). Flourishing is associated with many resources that likely lead individuals to perceive that they have the capacity to adapt to the demands associated with daily stressors (Keyes, 2002). The finding that people high in flourishing show lower stressor-related negative mood relative to those low in flourishing motivates future work to determine which of the many emotional, psychological, and social resources associated with flourishing (Huppert & So, 2013; Keyes, 2002, 2007) drive this finding.

The observation that people high in flourishing show reduced stressor-related negative mood relative to people low in flourishing provides insight into who tends to show weak versus strong associations between affect and stressor exposure. The repeated measures data also allowed us to consider when a given person may experience a weaker or stronger than usual association between negative mood and stressor exposure. Consistent with evidence that curiosity makes individuals more tolerant of uncertainty and distress, and less defensive when they experience uncertainty (Kashdan et al., 2013; Silvia, 2005), we found that today’s curiosity was a significant moderator of today’s stressor-related negative mood. More specifically, on days when curiosity was higher than usual, the association between today’s stressor exposure and negative mood was attenuated. Our operationalization of curiosity parallels research on reappraisal (Gunayden et al., 2016; Jamieson et al., 2013) and mindfulness (Langer, 1989); on days that participants were open to engaging with new and challenging experiences, their negative mood was lower in the face of stress.

Our curiosity findings are also consistent with prior research showing that mindfulness, often defined in terms of curiosity about the present moment (Bishop et al., 2004; Langer, 1989), attenuates stressor-related negative affect (Britton et al., 2012; Hoge et al., 2013). The assessment that curiosity contributes to mindfulness is longstanding. The Stoics, for example, insisted that philosophical curiosity affords a mindful perspective, while Theravada Buddhism identifies curious “investigation” (*dhamma vicaya*) as one of the seven factors of awakening. In contemporary scholarship, curiosity is often interpreted as a skill of openness to experience that produces mindful awareness (Williams, 2008). Conversely, sometimes mindfulness subscales are used to validate curiosity scales, given the latter’s correlation with an open and attentive attitude (Kashdan et al., 2009, 2013). Future work using mindfulness scales in tandem with curiosity scales will provide insight into the extent to which curiosity specifically drives previously observed associations between mindfulness and daily-stressor related negative mood.

We considered flourishing and day’s curiosity as separate moderators of the association between day’s stressor exposure and negative mood. Yet, curiosity may be a resource that people high in flourishing use to avoid higher negative mood following stressor exposure. Indeed, people high in flourishing demonstrate high levels of curiosity (Fredrickson, 2005; Fredrickson, 2001; Gallagher & Lopez, 2007; Lydon-Staley et al., 2019a), an association we also observe in the current sample (see Table 1). Thus, a clear direction for future longitudinal research is to test the extent to which robust experiences of daily curiosity mediate the association between flourishing and reduced stressor-related negative mood.

### 4.1 Limitations and Future Outlook

The findings should be interpreted in light of the study’s strengths and limitations. The use of daily diary data allowed us to capture fluctuations in negative mood, stressor exposure, and curiosity during “life as it is lived” (Bolger et al., 2003). However, daily diary data are limited in their ability to test causal associations. Indeed, our use of the term stressor-related negative mood rather than terms such as affective reactivity and recovery reflects the difficulty of capturing specific temporal stress-mood orderings using end of day daily diary reports (Stawski et al., 2019). Curiosity studies is a diverse field of research encompassing many definitions and operationalizations of curiosity (Zurn & Shankar, 2020). The findings of the present study are specific to notions of curiosity involving stretching and embracing components, indicating states involving the active seeking of opportunities for new experiences and a willingness to embrace the novel, uncertain, and unpredictable nature of everyday life (Kashdan et al., 2009). The extent to which they extend to other conceptions and operationalizations of curiosity as a feeling of deprivation to know more (Litman & Jimerson, 2004) or as a pleasurable feeling associated with discovering new information (Kashdan et al., 2018), for example, cannot be determined from the current data. We also note that we used a shortened version of an existing measure in order to reduce participant burden but that the shortened scale contained an item assessing the extent to which participants viewed challenging situations as opportunities to grow and learn.

## 5 Conclusions

In summary, we extend understanding of between-person and within-person factors associated with stressor-related negative mood. We find that flourishing is associated with attenuated daily stressor-related negative mood. We also find that states of curiosity are associated with diminished stressor-related negative mood. In doing so, we extend a large body of work indicating associations between stressor-related negative mood and psychopathology and poor physical health to trait and state markers of well-being.

## Figures and Tables

**Table 1:** Correlations and descriptive statistics

Variables	1	2	3	4	5
1. Flourishing	–				
2. Daily curiosity^a^	0.25*	–	– 0.18***	– 0.06***	
3. Daily negative mood^a^	– 0.30***	0.03	–	0.24***	
4. Daily stressor exposure^a^	0.09	0.07	0.43***	–	
5. Age	– 0.07	0.05	– 0.06	0.08	–
Variables					
Mean	5.92	3.09	1.75	0.84	25.37
Standard deviation	0.80	1.85	1.25	0.20	7.32

*** *p* < 0.001

* *p* < 0.05. A subset of these correlations has been reported in previous work (Lydon-Staley, Zurn, et al., 2019)

a Values on the lower triangle indicate correlations between the intraindividual mean of the daily diary time series to capture between-person differences in average daily curiosity, negative mood, and stressor exposure; values on the upper triangle indicate repeated measure correlations of the time series data to capture within-person associations. N=167

**Table 2:** Results of the multilevel model examining flourishing and daily curiosity’s moderating effects on the association between today’s stressor exposure and negative mood

Fixed effects			
	Estimate	Standard error	*p*-value
Intercept $\left(\gamma_{00}\right)$	1.02***	0.08	<0.001
Today’s stressor exposure $\left(\gamma_{10}\right)$	0.85***	0.08	<0.001
Today’s curiosity $\left(\gamma_{20}\right)$	−0.02	0.03	0.42
Flourishing $\left(\gamma_{01}\right)$	−0.22*	0.09	0.01
Usual stressor exposure $\left(\gamma_{02}\right)$	0.97**	0.32	0.003
Usual curiosity $\left(\gamma_{03}\right)$	0.06	0.04	0.08
Flourishing × today’s stressor exposure $\left(\gamma_{11}\right)$	−0.23*	0.09	0.01
Today’s curiosity × today’s stressor exposure $\left(\gamma_{21}\right)$	−0.10***	0.03	< 0.001
Random effects			

	Estimate	95% Confidence intervals	
Intercept $\left(\sigma_{u0}^{2}\right)$	0.56	0.42–0.75	
Today’s stressor exposure $\left(\sigma_{u1}^{2}\right)$	0.54	0.40–0.74	
Today’s curiosity $\left(\sigma_{u2}^{2}\right)$	0.12	0.09–0.15	
Correlation $\left(r_{u0u1}\right)$	0.98	−0.11–0.99	
Correlation $\left(r_{u0u2}\right)$	−0.24	−0.63–0.24	
Correlation $\left(r_{u1u2}\right)$	−0.18	−0.60–0.32	
AR1	0.20	0.16–0.24	
Residual	1.01	0.98–1.04	
Fit indices			
AIC	9397.83		
BIC	9494.59		

N = 3134 days nested within 167 participants. Flourishing was sample-mean centered

## Data Availability

Data are available at this Open Science Foundation link: https://osf.io/kjz7g/.
